# Archaeometric Characterization of the Industrial Production of Porcelains in the Vieillard & Co. Manufactory (Bordeaux, France, 19th Century)

**DOI:** 10.3390/ma15155311

**Published:** 2022-08-02

**Authors:** Emmie Beauvoit, Nadia Cantin, Quentin Lemasson, Rémy Chapoulie, Ayed Ben Amara

**Affiliations:** 1 Archéosciences Bordeaux, UMR 6034 CNRS, Université Bordeaux Montaigne, Université de Bordeaux, Maison de l’Archéologie, Domaine Universitaire, Esplanade des Antilles, CEDEX, 33607 Pessac, France; ncantin@u-bordeaux-montaigne.fr (N.C.); chapouli@u-bordeaux-montaigne.fr (R.C.); ayed.ben-amara@u-bordeaux-montaigne.fr (A.B.A.); 2 Centre de Recherche et de Restauration des Musées de France (C2RMF), Palais du Louvre, 14 quai François Mitterrand, 75001 Paris, France; quentin.lemasson@culture.gouv.fr; 3 FR3506 New AGLAE, CNRS/Ministère de la Culture, 14 quai François Mitterrand, 75001 Paris, France

**Keywords:** porcelain, 19th century, Vieillard & Co. manufactory, SEM-EDS, PIXE-PIGE, XRD

## Abstract

In this paper, we focus on the industrial production of porcelain in the Bordeaux area (France) in the 19th century. Our main objective is to assess the evolution of production technology of the same manufactory over a period of more than 40 years. A multi-analytical approach was used to investigate glazes and bodies of thirty-four sherds of biscuit and porcelain found in an archaeological context. The microstructural, chemical, and mineralogical characterization was performed using a combination of scanning electron microscopy, coupled with energy dispersive spectroscopy (SEM-EDS), particles induced X-ray and gamma emission (PIXE-PIGE), and X-ray diffraction (XRD). Results obtained on the characterization of the ceramic production technologies and on the chemical modification over time contributes to investigate this industrial production, which is not well documented by the written archives. The examination of the biscuits, rare artifacts, showed that the porcelain bodies were produced by mixing kaolinitic clays, quartz, and potassium feldspars. The mineralogical analysis of the ceramic supports allowed hypotheses to be put forward on the temperatures of the biscuit firing (around 950 °C) and the second firing (over 1200 °C). Furthermore, the treatment of the compositional data, including both glazes and bodies, using multivariate statistical analysis, revealed different types of production corresponding to the different chronological periods of production at Bordeaux throughout the 19th century. These results will enable us to consider the possibility of authenticating non-stamped and undecorated pieces.

## 1. Introduction

During several decades of the 19th century, between 1850 and 1895, the French Vieillard & Co. manufactory produced porcelains, among other things. Unfortunately, only few objects from this production remain in museums or private collections. Recently, through archeological excavations in Bordeaux (France) performed in 2015 by the Centre Archéologie Préventive de Bordeaux Métropole, waste dumps of the Vieillard & Co. factory were discovered and have provided significant quantities of porcelain fragments [1]. Before this archeological discovery, the importance of porcelain production in Bordeaux remained practically unknown [2]. As a matter of fact, the quantity of pieces produced was significantly underestimated. The reasons are multiple and include the fact that: (1) in most cases, porcelain is not decorated, and (2) it would seem that porcelain generally does not have a stamp. In addition, the estimation of the productions was distorted by selective conservation, which has been the result of time and collections, often limited to beautiful pieces. Furthermore, manufactory archives (that were lost or destroyed) are severely lacking in order to document the production at the factory (especially regarding the choice of raw materials, recipes, firing conditions, division of labor, and life in the manufactory, etc.). Consequently, archeological excavations in the ancient factory area and representative sample collection have made it possible to reevaluate the diversity of the objects produced by the factory. In addition, this discovery gave us the opportunity to apprehend production organization and manufacturing processes of the Vieillard & Co. manufactory. This industrial factory was founded in Bordeaux in 1845. Jules Vieillard produced a wide variety of decorative and utilitarian products: white earthenware, hard-paste porcelains, and bottles of glass. The two sons of J. Vieillard took over the factory in 1865, just before the death of their father (1868). When the two brothers died—in 1893 and 1895, respectively—the company closed and never reopened its doors again [2]. 

As it has already been illustrated, the chemical and mineralogical analysis of archaeological porcelain artifacts can provide insights into the firing protocol, the recipess, or the provenance of raw materials [3,4]. The present research focuses on the technical evolution of the porcelain productions during the different stages of the Vieillard factory life. For the first time, these data—provided by manufactory waste dumps—allow us to understand the production evolution of the same French manufactory over a period of more than 40 years. Actually, excavations revealing 19th century factory dump remain very scarce. The main objective of this paper is to make assumptions about production techniques (the ceramic body preparation process and firing conditions) and to identify recipe evolutions over time. For this purpose, archaeological sherds (both the body and the glaze) were characterized involving chemical (SEM-EDS and PIXE-PIGE) and structural (XRD) analysis techniques. Another goal discussed in this work is to determine if it is possible to differentiate the different productions of the Vieillard & Co. factory. To address this issue, we will rely on the extensive and comprehensive dataset acquired on the chemical composition of archaeological porcelain from this industrial plant. Finally, in a wider context, this work focuses on the bodies and the glazes of Vieillard & Co porcelains, which will be helpful for the authentication of undated objects and for comparisons with other European productions from the same chronological period.

## 2. Materials: Archaeological Sherds

In 2015, the historic waste dumps of the factory were discovered during excavations in the Bacalan district in Bordeaux. This excavation provided significant quantities of pottery production waste pertaining to the different stages of the *chaîne opératoire* of ceramics fabrication (raw materials, plaster molds, kiln furniture, pigments, biscuits, and glazed white earthenware). The archeological materials can be dated by archaeologists through the analysis of stratigraphy and stamps printed on the back of some white earthenware pieces, as described by Marache et al. [1]. Chronological ranges can thus be defined, and for ease of reading, in this article, the different periods are presented by estimated dates, as summarized in Table 1.

**Figure 1:**
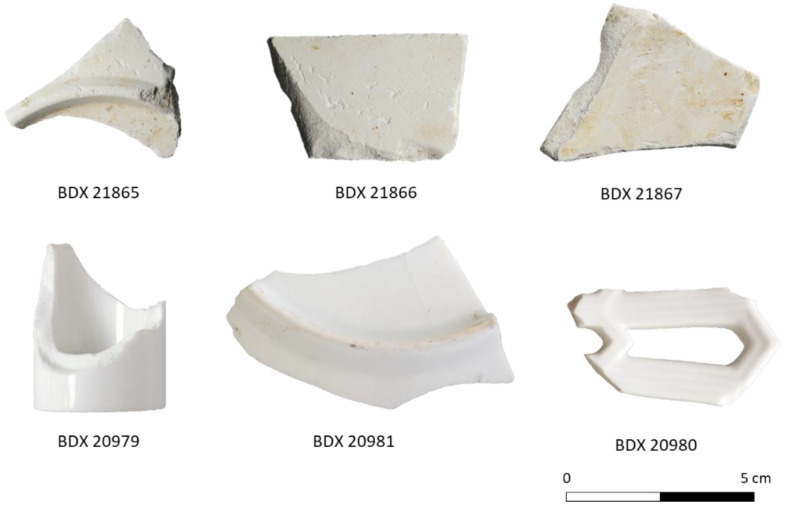
Some examples representative of the analyzed samples: biscuits of porcelain at the top and porcelains at the bottom.

In the present study, we consider three groups of sherds that correspond to the different production periods. These sherds come from the available and abundant artifacts found during the excavation located in the ancient waste dumps of the manufactory. A total of thirty-four fragments were sampled: twenty-eight porcelains and six biscuits of porcelain (Figure 1). In some cases, their dimensions allow us to recognize the shape and the use of the original objects.

## 3. Analytical Techniques

### 3.1. Archaeological Samples Preparation

Cross sections of the porcelains were prepared to study the ceramic microstructure using scanning electron microscopy (SEM) and chemical composition using energy dispersive spectrometer (EDS) and particle induced X-ray and gamma emission (PIXE-PIGE). Samples were prepared by coating the porcelain cross-sections in resin (Araldite^©^ 2020). After drying, samples were ground to mirror finish using a series of lapping wheels, with diamond grits from 6 to a 1 µm grit. Another sample is necessary to realize the mineralogical analysis. Therefore, each sample was powdered in a planetary mill using tungsten carbide cells. Before grinding, glazes and exterior surfaces were mechanically removed in order to minimize contamination from glaze and soil into the sample.

### 3.2. Scanning Electron Microscopy-Energy Dispersive X-ray Spectroscopy (SEM-EDS)

The bulk chemical composition of the bodies and glazes was investigated through polished cross sections using scanning electron microscopy-energy dispersive X-ray spectroscopy (SEM-EDS) (JEOL JSM-6460 with EDS, Oxford X-Max 20) in low-vacuum mode. The operating measurements were 20 kV acceleration voltage, 10 mm working distance (WD), and about 500,000 counts per spectra. Micrographs were recorded using the back-scattered electron mode (BSE). The chemical compositions of the bodies were determined by analyzing a minimum of three areas of approximately 1.0 × 0.6 mm^2^. The analyses were normalized to 100 wt% and then averaged. Quantification was determined using the ϕ (ρz) correction procedure for the INCA software (Oxford Instrument, United Kingdom). Standard corrections were performed using the software’s internal standard, and contents were calculated from standards consisting of synthetic compounds and natural minerals. The detection limit for most elements was about 0.1 wt%.

### 3.3. X-ray Diffraction (XRD)

The identification of the porcelain body powder crystalline phases was carried out with an X-ray diffractometer D8 Discover (Bruker, USA) by using Cu-Kα radiation generated by an accelerated voltage of 40 kv and a filament current of 40 mA. The samples were scanned from 3–60° 2θ in a step size of 0.02° 2θ. Data were analyzed using the DIFFRAC. EVA software (Bruker AXS), and the diffraction patterns of the samples were analyzed using a search matched against the International Center for Diffraction Data (ICDD) database.

### 3.4. Particle Induced X-ray and Gamma Emission (PIXE-PIGE)

The bulk and glaze chemical composition of porcelains was also non-destructively investigated using the NewAGLAE ion-beam accelerator in the Centre de Recherche et de Restauration des Musées de France (C2RMF) (description can be found in Pichon et al., 2014) [5]. PIXE and PIGE spectroscopies were simultaneously performed using a 3 MeV proton beam. PIXE analyses were performed with four X-ray SDD detectors, using one with a helium flux to measure the light elements and for the other three with a 50 µm Al filter to measure the heavy elements. PIXE analysis was used to determine major and minor oxides (SiO_2_, Al_2_O_3_, Fe_2_O_3_, MgO, CaO, K_2_O, TiO_2_, expressed as concentrations in oxide weight %) and trace elements (Mn, Cu, Zn, Ga, Rb, Sr, expressed in ppm). Simultaneously, PIGE was performed using a high purity germanium (HPGe) radiation detector and quantified sodium (Na_2_O). GUPIX software was used for PIXE analysis to obtain elemental concentrations. The relative error for concentration values is about 1% for major and minor elements and 5% for traces [6]. Each analysis was performed by scanning the 30 µm-diameter-beam across one 2000 × 1000 mm^2^ area in order to obtain an average composition of the sample that may be inhomogeneous. In order to ensure the correctness of the data and to make sure that the accelerator produced accurate concentrations, the same standard specimens were used during each run (DrN, MaN, SV4001, and SV4002).

## 4. Results and Discussion

### 4.1. Body Characterization

#### 4.1.1. Biscuits of Porcelain

At first glance, very few porcelain biscuits were found during the archaeological excavations. Among the thousands of white earthenware biscuits found, only six have been identified as porcelain biscuits. The reasons for this remain unknown, but it could be explained by the recycling of the pieces which would have otherwise been thrown away because of their non-conformity. Furthermore, some ancient texts suggest that the biscuits can be crushed to be recycled and added to the ceramic body [7,8]. Moreover, it would seem that this kind of addition is not limited to biscuits, but also concerns glazed porcelain sherds, which can be added to the body recipe in quantities up to 10% [9].

Through observation with the naked eye and a binocular magnifying glass, one can distinguish a friable texture, smooth to the touch, which can be easily scratched, like plaster (Figure 1). The first firing stage solidifies the porcelain so that it can be handled without breaking; at this step, the piece is still porous [10]. 

**Figure 2:**
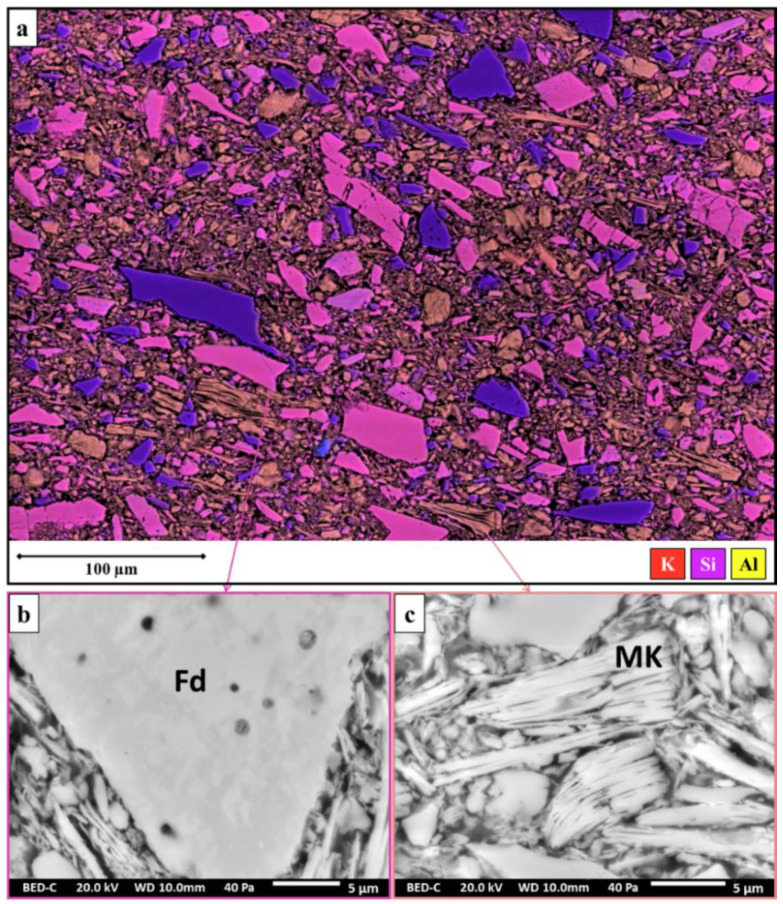
(**a**) Elemental mapping of biscuit body obtained by SEM-EDS and SEM backscattered electron images of (**b**) K-feldspar (Fd) and (**c**) coarse meta-kaolinite flake (MK) present in biscuit.

The SEM-EDS study of the six porcelain biscuit sherds allowed us to hypothesize about the different ingredients used in the fabrication of the clay supports. The observations of the body’s section with SEM reveal the presence of at least three types of inclusions, whose granulometry does not exceed 100 µm, which could be identified due to their microstructure and their chemical composition. The X-ray mapping obtained by SEM-EDS allows to visualize the distribution of the main chemical elements present, namely silica, aluminium, and potassium (Figure 2).

The results show that the first phase identified is particularly rich in silica (more than 98 wt% in SiO_2_) and could be related to quartz. A second type of inclusion contains mainly silica, aluminium, and potassium (respectively, up to about 68 wt% in SiO_2_, 21 wt% Al_2_O_3_, and 9 wt% K_2_O), which could corroborate the addition of potassium feldspars in the ceramic body. Finally, the last inclusion, recognizable by its stacked platelet structure, is chemically characterized by high silica and aluminum contents (about 55 wt% SiO_2_ and 45 wt% Al_2_O_3_, respectively). These data suggest the presence of metakaolinite (an amorphous compound with chemical formula Al_2_Si_2_O_7_) [11]. The latter results from the transformation of kaolinite (added as an ingredient in the form of kaolinitic clays) during the firing of the ceramic body. The reaction sequence of kaolinite calcination allows us to estimate that kaolinite decomposes into metakaolinite, starting at about 550 °C, before disappearing at around 950 °C [12,13]. The analysis of the six biscuits allows us to describe the main ingredients used in the production of porcelain bodies: quartz (introduced in the form of sand, for example), potassium feldspars, and kaolin. These ingredients are the basis of the recipes for hard porcelain made in the 19th century [7].

EDS analysis of the porcelain biscuits indicates the abundant presence of silica (more than 64 wt% as SiO_2_, on average) and aluminum (about 29 wt% as Al_2_O_3_) (Table 2). On the other hand, it is noted that the K_2_O content is about 5 wt% on average, while the Na_2_O concentration is relatively low (0.8 wt% on average). Calcium and iron are present in small amounts (0.2 and 0.3 wt% in CaO and Fe_2_O_3_, respectively). These compositional data confirm the presence of the potential ingredients identified previously.

The structural analysis carried out on the six biscuits confirms the presence of quartz (SiO_2_) and potassium feldspars (in the form of microcline, KAlSi_3_O_8_). Some samples (*n* = 3) also contain a small amount of mullite (3Al_2_O_3_·2SiO_2_), formed from kaolinite via metamorphosis at around 1200 °C [14]. This result, associated with the presence of metakaolinite in the support, allows us to put forward the hypothesis that the biscuit of the porcelain was fired at about 950 °C. This temperature would explain the coexistence of metakaolinite (visible in the SEM) and mullite, whose peaks appear on the diffractograms. This approximate temperature of the biscuit firing is in agreement with the literature [10,15]. Although, as Alexandre Brongniart indicates, for hard porcelain, the first firing, or “biscuit, is only intended to firm up the paste enough to make it easier to give it the glaze by immersion; but we could do without it, and fire the ceramic body and the glaze at the same time, as they require the same temperature” [7] (p. 256).

#### 4.1.2. Porcelains

**Figure 3:**
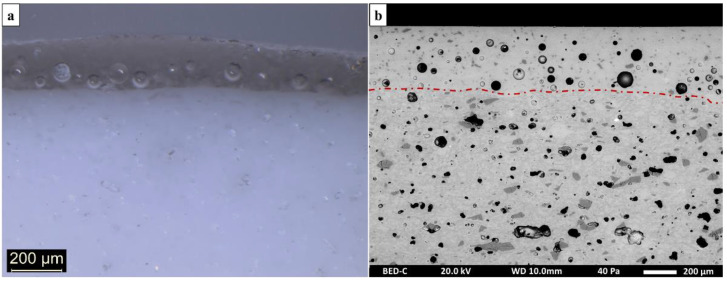
Microphotograph (**a**) and SEM backscattered electron image (**b**) of a porcelain sample. The red dotted line marks the boundary between the glaze and the ceramic support.

**Figure 4:**
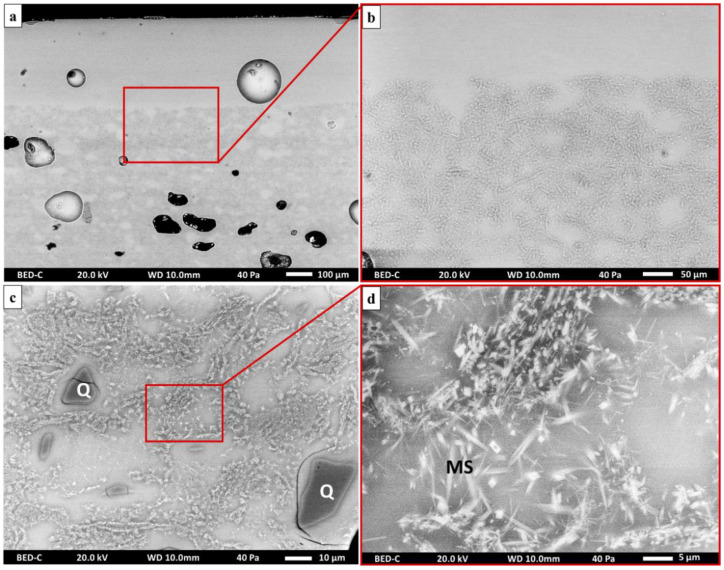
SEM backscattered electron images of the porcelain body (**a**,**b**); the presence of secondary mullite crystals (MS) and quartz (Q) can be noted (**c**,**d**).

Observations made with optical microscopy on the porcelain samples showed that the samples have a white ceramic support covered with a transparent glaze (Figure 3a). At first glance, the distinction between the ceramic support and the glaze is complex to apprehend with SEM (Figure 3b). Nevertheless, several clues allow for the visualization of the limit of the two components: first, numerous bubbles remain in the glaze, especially at the interface with the ceramic support, as observed under white light; second, the abundance of crystals is visible exclusively on the supports and absent in the glazes (Figure 4).

These crystals of acicular shapes and micrometric granulometry, are identified by SEM as secondary mullite (Figure 4d) [16,17]. This hypothesis is in agreement with the use of kaolinitic clays, whose calcination generates the appearance of secondary mullite from about 1050 °C [12,13]. In addition, in the ceramic supports, we also observe inclusions composed almost exclusively of silica, probably quartz, whose granulometry varies between 10 and 50 µm and which would result from an incomplete dissolution of the raw materials.

The ceramic support is characterized by a large number of closed pores due to the high viscosity of the melt, so that the gases emitted during firing could not escape (Figure 4a) [18]. Although the sherds do not show any notable difference, it could be interesting to quantify, by image processing, the porosity induced by firing to compare these productions with others that are contemporary to them [19,20]. In this case, the unoriented pores appear in various forms: elongated, semi-rounded, and deformed. This observation suggests that molding and casting techniques were used to make the ceramic pieces, which is supported by the abundant plaster mold finds at the manufactory dump site [1].

The chemical composition of the ceramic supports of the porcelains was determined by SEM-EDS for the whole corpus (concerning major and minor elements), as well as by PIXE (performed on 19 sherds in order to identify and quantify additional trace elements). All the chemical compositions are shown in the Supplementary Material (Tables S1 and S2), while the average values for each defined chronological group are reported in Table 3 and Table 4.

SEM-EDS analysis reveals that all ceramic supports are predominantly composed of silica (SiO_2_ contents above 64 wt%) and aluminium (Al_2_O_3_ contents between 24 and 30 wt%). The bodies are non-calcareous (<0.7 wt% in CaO) and contain a low iron concentration (<0.4 wt% in Fe_2_O_3_), which confirms the whiteness of the porcelain body. The fluxes used are alkaline, rich in potassium (between 3.3 and 4.6 wt% in K_2_O) and sodium (1.3 and 2.4 wt% in Na_2_O), (Table 3).

The main variations in chemical composition are observed between the production group of Jules Vieillard (c. 1855) and the two groups of his sons. These chemical variabilities (an increase in K_2_O content and a decrease in Na_2_O content) concur, from a temporal point of view, with the takeover of the factory by Albert and Charles Vieillard. However, it is important to emphasize that the small number of samples induces relatively large standard deviations, which complicate their interpretation and thus, the certain attribution to a given group.

**Figure 5:**
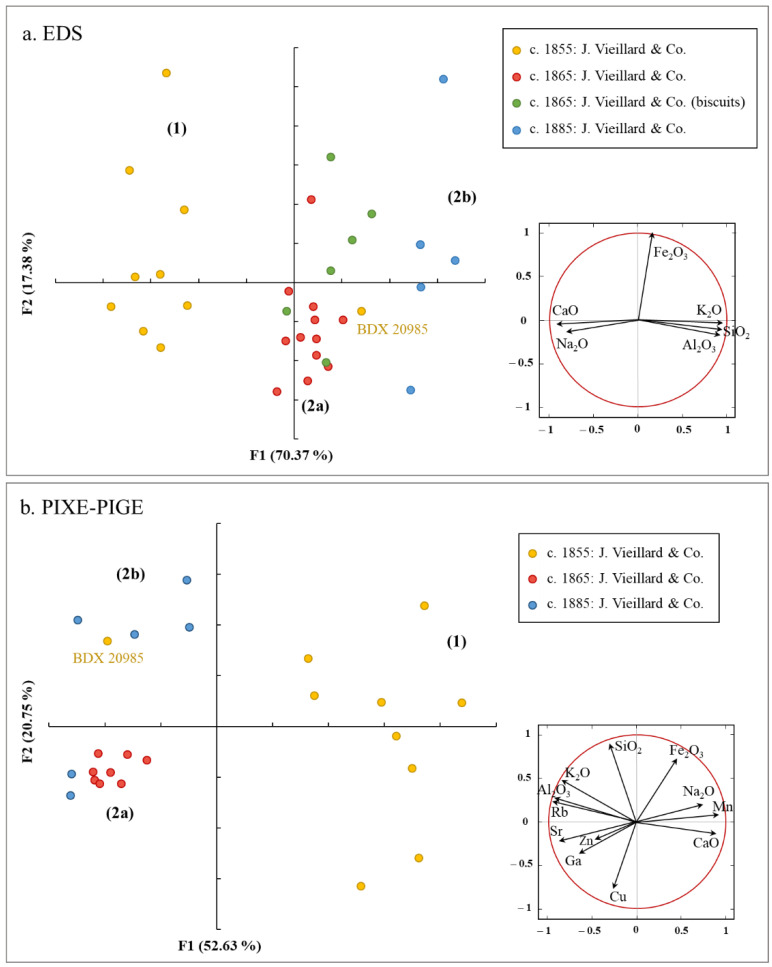
PCA score/loading plot obtained with (**a**) EDS and (**b**) PIXE-PIGE data of some detected elements of the porcelain bodies.

Principal component analysis (PCA) after transformation into centered log-ratio (clr) of the concentrations obtained by SEM-EDS (Na_2_O, Al_2_O_3_, SiO_2_, K_2_O, CaO, and Fe_2_O_3_) allows for the visualization of the projection of the individuals and variables [21]. This representation shows a division of the samples into two distinct groups, divided as follows: (1) the Vieillard & Co production (c. 1855), characterized by high Na_2_O (2.4 wt% on average) and CaO (about 0.7 wt%) contents, and (2) the Vieillard sons productions, which are distinguished by higher Al_2_O_3_ (more than 27.5 wt% on average) and K_2_O (more than 3.9 wt% on average) concentrations and lower SiO_2_ (less than 66 wt%) concentrations (Figure 5a). At this stage, and because of the limited number of samples analyzed, it appears difficult to discriminate, from a chemical point of view, these two phases of porcelain production (c. 1865 and c. 1885). However, we note that these distinctions seem to be divided into two subgroups: (2a) centered on the period around 1865, and (2b) centered on the period around 1885, discriminated according to the concentration of K_2_O, which increases over time. Only sample BDX 20985 (dated to the production period of the father, Jules Vieillard) does not appear to belong to the chronological group to which it was archaeologically related. Furthermore, a comparison of the chemical compositions of the porcelain bodies and those of the biscuits corroborates the identification that has been made of these sherds. Actually, the group of the biscuits (c. 1865) overlap with the group of porcelains corresponding to the same chronological period.

The PIXE data allow us to confirm the identification of at least two chemically distinct groups (Figure 4b). The samples associated with the productions made under Jules Vieillard and those related to the Vieillard sons are clearly separated. As we have just seen with the EDS data, one can also wonder if the two productions of the sons could not be split into two groups of different chemical compositions. Nevertheless, this conclusion deserves to be supported by analyzing more samples from the sons’ production periods.

The contents of major and minor elements are comparable between SEM-EDS and PIXE. Regarding the traces detected, several results are worth highlighting. Thus, although the differences between the values are tenuous, we observe that the ceramic bodies of the productions of Jules Vieillard (c. 1855) contain a high concentration of manganese (about 80 ppm), while the productions of the sons present high contents of rubidium (around 280 ppm on average). This concentration could be correlated to the higher contents of potassium, and thus to the presence of potassic feldspars (Table 4).

**Figure 6:**
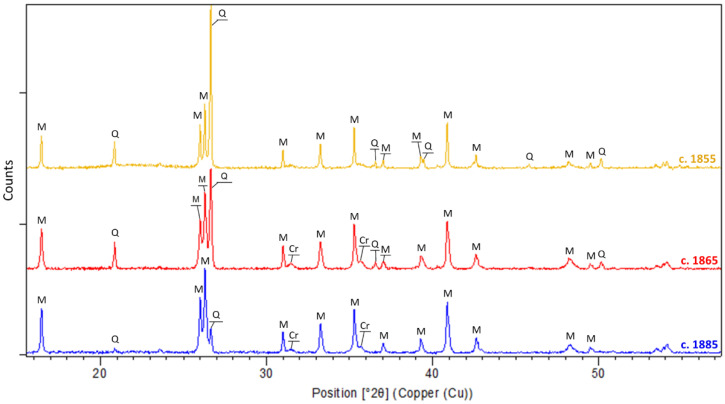
Diffractograms of a representative sample of porcelain bodies for each identified production period; Q: quartz, Cr: cristobalite, M: mullite.

Nineteen of the porcelain clay supports were analyzed by X-ray diffraction. The diffractograms reveal the presence, in all the samples of the corpus, of an amorphous phase highlighted by an elevation of the signal background. The identification of this amorphous phase is consistent with the vitreous nature of the porcelain body. The main mineralogical phases identified in all the ceramic supports are mullite (3Al_2_O_3_·2SiO_2_), quartz (SiO_2_), and cristobalite (SiO_2_) (Figure 6). The presence of these phases corroborates the data acquired previously, both in elemental composition (the clay supports contain more than 64 wt% in SiO_2_ and more than 24 wt% in Al_2_O_3_) and according to SEM observations (the acicular crystals identified as secondary mullite).

As previously discussed, the presence of secondary mullite in the bodies indicates that the firing temperature is at least 1050 °C [12,13]. Furthermore, the absence of potassium feldspars (identified in porcelain biscuits) allows us to specify the temperature range for the second firing of the pieces. Feldspars play the role of fluxes to bind together the different components of the paste; they therefore participate in the formation of the glassy phase. As shown in a study characterizing porcelain made from the previously identified elements (kaolin, quartz, and feldspars), the feldspars contained in the paste decompose and melt above 1230 °C [16]. This hypothetical minimum firing temperature is consistent with ceramic treatises that attest to the firing of glazes on hard porcelains between 1280 and 1400 °C [10,15,22]. 

The firing of hard-paste porcelain is a key point of its production, because its price depends mainly on this step. Indeed, the firing of hard porcelain makes it an expensive product, essentially because of the high cost of wood, which must be used in large quantities to reach the desired high temperatures [23]. At the Vieillard & Co factory—as in other contemporary factories—they tried to lower the cost price of firing and therefore, of porcelain, by using coal rather than wood because at the time, “Everyone knows that for the same caloric value, wood is much more expensive than coal everywhere in France” [23] (p. 247). In this regard, it is noteworthy that the first coal firing took place at the end of the 18th century in Paris [23]. In Bordeaux, the first trials of coal firing were set up by Jules Vieillard and are explained in detail in his correspondence with Joseph Ebelmen (director of the Sèvres Manufactory); Jules Vieillard explains that he used “a new kiln of 18 feet in diameter and 13 and a half feet in height. The total duration of the fire is, on average, 36 to 40 h for the small, medium and large fire. He employs for labor during these 40 h of cooking from 5 to 6 men for the care of the fire—the last one is called only when the coal is of inferior quality and requires constant scraping of the grates. The use of coal is 18 to 20,000 kilos per batch” (Archives of the Sèvres Manufactory, series U20, file 19, handwritten letter from Jules Vieillard to Joseph Ebelmen, director of the Sèvres Manufactory, dated 15 September 1851). In his report, Alphonse Salvétat highlights that the production of hard porcelain in the Bordeaux factory depends largely on this technical choice that was made: “The use of the mineral fuel, introduced in the Manufactory of Bordeaux by the perseverance of the director makes him the greatest honor. The manufacture of hard porcelain in this establishment was impossible in any other condition. The high price of vegetable fuel in the locality could not allow to fight advantageously with the existing factories. It is known that the value of the fuel represents about the third of the price of the hard-paste porcelain” (Archives of the Sèvres Manufactory, Qb26, Report made to the Société d’Encouragement by Mr Salvétat, on behalf of the Bordeaux Commission and the Chemical Arts Committee, January 1855).

Although the phases identified in the porcelain body are the same in all samples, it should be noted that the ratio of quartz (Q) to mullite (M) peaks decreases throughout the production period (Figure 6). As a matter of fact, the diffractograms show an increase in the quantity of mullite compared to quartz in the supports. This evolution could be explained by an increase in the amount of kaolin added [24], or by a change in the firing protocol (step and temperature) [12]. However, this observation would deserve further investigation with the implementation of Rietveld analyses [25].

The study of the ceramic supports of the archaeological sherds having enabled us to distinguish the various periods of production of the manufactory of Bordeaux, we will now focus more specifically on the glaze. Actually, this last one constitutes the only source of information available for non-destructive investigation.

### 4.2. Glaze Examination

**Figure 7:**
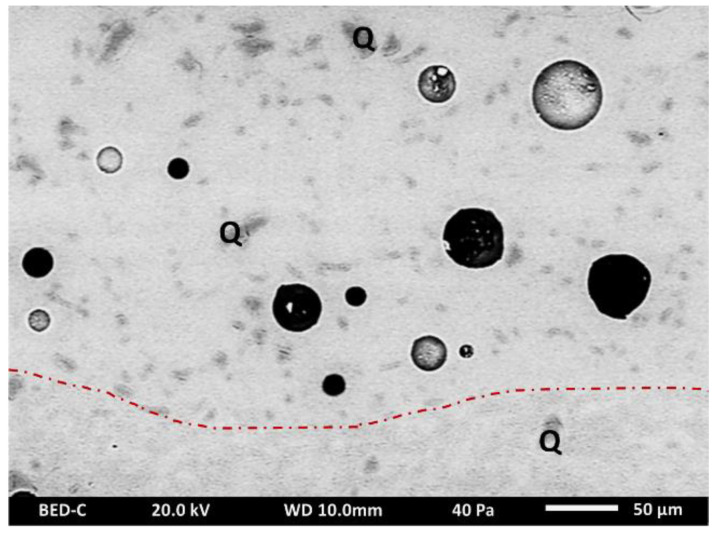
SEM backscattered electron images of porcelain glaze; the presence of quartz (Q) in the glaze (at the top of the red dotted line) and in the body (at the bottom of the red dotted line).

All the fragments studied have a transparent, colorless glaze (Figure 3a). SEM observation of the glazed porcelains reveals a large number of bubbles within their glaze. These bubbles, ranging from 10 to 100 µm in diameter, are the result of the decomposition of the raw materials of the glaze mixture, and possibly the biscuit, during firing. The thickness of the glazes varies between 50 and 300 μm on all samples in the corpus. The glaze is also characterized by the presence of dispersed inclusions of variable granulometry (between a few micrometers to less than 50 µm) (Figure 7). These unmelted inclusions, mainly composed of silica (more than 98 wt% in SiO_2_), are identified as quartz grains. They could be the result of the glaze mixture, or even of the decomposition of the ceramic support during the second firing.

The porcelain glazes were analyzed using two analytical techniques: SEM-EDS and PIXE. The first method allowed for the determination of major and minor elements for all the sherds in the corpus, while the PIXE allowed the detection and quantification of major, minor, and trace elements present in the glazes of 19 sherds. The complete results for each sample are presented in Supplementary Material (Tables S3 and S4).

The chemical analysis conducted with SEM-EDS on the whole corpus reveals that all glazes are mostly composed of silica (SiO_2_ contents higher than 74 wt%) and aluminium (Al_2_O_3_ contents between 13 and 16 wt%). In addition, the glazes contain alkaline fluxes: potassium (between 4.1 and 5.5 wt% in K_2_O on average) and sodium (between 1.6 and 3.3 wt% in Na_2_O). Finally, the presence of calcium (between 0.5 and 1.7 wt% in CaO) and the low content of Fe_2_O_3_ (0.3 wt% at the most) are noted (Table 5).

Although the contents within the corpus do not change significantly, it is interesting to note that the proportions of fluxes evolve over time: the concentrations of K_2_O increase, while the amount of sodium decreases. Similarly, the CaO content also declines with time. It is also interesting to point out here that these same trends were observed for the ceramic substrates. Simultaneous firing of the body and the glaze requires that there be an agreement between the vitrification temperature of the paste and the firing temperature of the glaze [22]. Therefore, it can be hypothesized that there was an adjustment in the chemical composition of the paste and glaze, particularly in terms of the coefficient of expansion.

**Figure 8:**
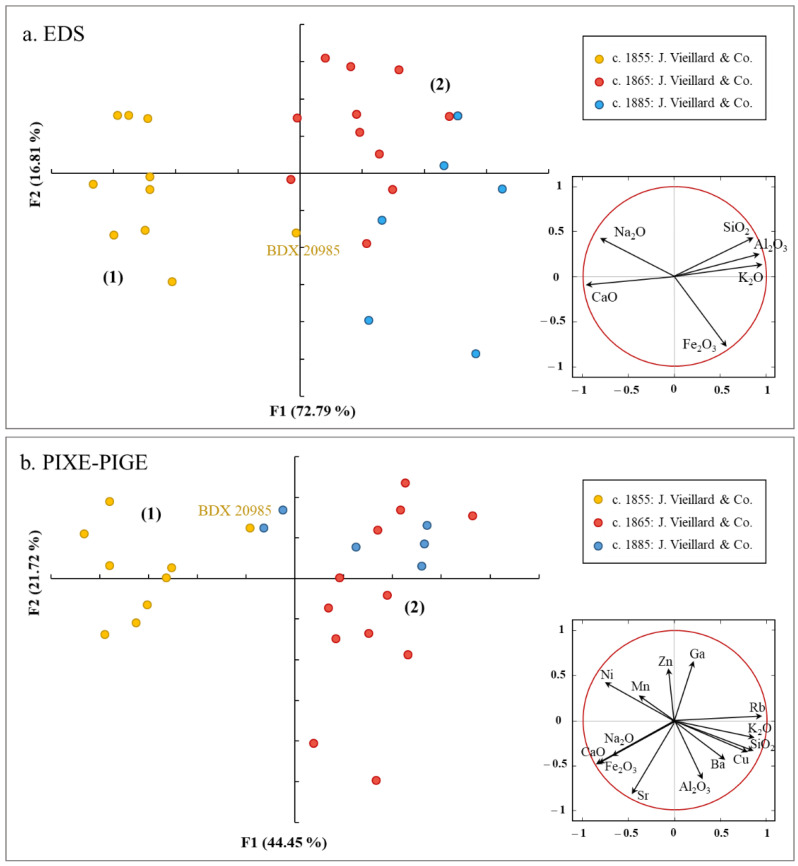
PCA score/loading plot obtained with (**a**) EDS and (**b**) PIXE-PIGE data of some detected elements in the glazes.

Principal component analyses (PCA) of the glazes, produced from the SEM-EDS and PIXE data, reveal two distinct groups (Figure 8a,b). The chemical results allowed us to conclude the distinction of two compositional groups: (1) the father’s production (c. 1855) and (2) the sons’ production (c. 1865 and c. 1885). The first group seems relatively homogeneous, except for sample BDX 20985, whose chemical composition is close to the second group (as was the case for the composition of the ceramic body). This piece is perhaps the artifact of a test and/or the witness of a transition accompanied by a change in recipes or raw materials. The second group, composed of sherds from Vieillard’s productions, is relatively dispersed. 

From the chemical elements detected in the glazes, some hypotheses can be made about the ingredients used. The significant presence of SiO_2_ suggests that quartz was added to the glaze recipes. The addition of feldspars could explain the presence of silica, aluminum, potassium, and sodium. As for calcium, which is not abundantly present in the productions of Vieillard’s sons, it could have been added in the form of chalk [7]. Nineteenth century treatises indicate the use of a single ingredient to make the glaze: pegmatite [7,22]. This rock, also called “pebble,” provides all the elements necessary for the formation of the glaze: “In France, it is used almost exclusively as a glaze of the pegmatite of Saint-Yrieix more or less decomposed and which, in this state, is a mixture of feldspar, quartz and kaolin. One can regulate its fusibility by adding a certain quantity of quartz” [22] (p. 703).

The trace elements, detected by PIXE, also provide valuable information for discriminating these productions. The presence of nickel and manganese is characteristic of the first period of production identified (around 1855). On the contrary, the productions of the sons of Jules Vieillard are marked by higher contents of rubidium and copper (Figure 8b and Table S4). These differences may reflect a change in the provenance of raw materials. Furthermore, the written texts, although sparse, allow us to apprehend the various locations supplying raw materials (especially in feldspars and kaolinitic clays) for the factory. In 1839, a section of the report of the Exhibition of Products of French Industry is devoted to the Vieillard manufactory, which exhibited some of its pieces. It is mentioned that the feldspar and kaolin employed “usually come from St-Yrieix near Limoges, but they also imported them from England” (Departmental Archives of the Gironde, 8M91, Report of the Departmental Jury of the Exhibition of the products of the French industry of 1839.). From 1843 onwards, “the factory gets most of its clay from the department; it also collects it from neighboring departments, and for some products it imports kaolin from the Pyrenees” [26] (p. 351). This archival investigation was followed by a geological prospection in the Pyrenees in order to identify and locate extraction sites. In the future, the chemical study of the geological material collected on-site may allow us to verify if there is a correlation between the chemical signature of the porcelain and the clay used. 

### 4.3. Comparison with Other European Hard-Paste Porcelain Productions 

Few archaeometric studies on porcelains (body and glaze) of the 18th century are available in the literature. We consider published data on European hard-paste porcelain for comparison with data obtained on productions from the Vieillard & Co manufactory. These published works focus on hard porcelains from museums and excavations in consumption sites [27,28,29,30,31,32,33]. In these studies, productions of the Saxon manufactory of Meissen [27,28,29,30,31], an Italian manufactory (Vinovo) [32], and two English factories (Bristol and Plymouth) [33] were chemically characterized. These compositional data were obtained by SEM-EDS [27,28,29,32], portable X-ray fluorescence [30,33], or PIXE-PIGE [31] analysis. The results were compared with EDS data acquired from production samples from Vieillard & Co factory, without using a correction factor.

**Figure 9:**
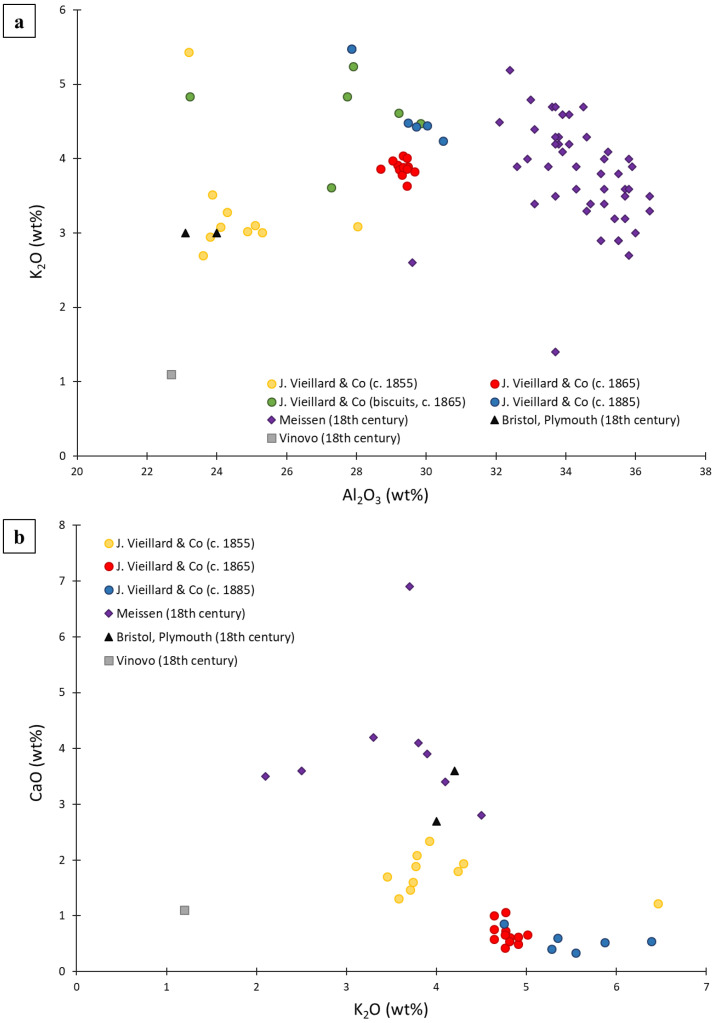
Bivariate plots of (**a**) K_2_O vs. Al_2_O_3_ to compare the composition of porcelain bodies of Vieillard & Co measured by EDS to other European porcelains and (**b**) CaO vs. K_2_O to compare the composition of porcelain glazes of Vieillard & Co measured by EDS to other European porcelains. The compositional data used for comparisons were published in Schulle and Ullrich, 1982; Ullrich et al., 2010; Domoney et al., 2012; Bezur and Casadio, 2009; Neelmeijer et al., 2014 (for the Saxon manufactory of Meissen), in Tite and Bimson, 1991 (for two English factories) and in Turco et al., 2016 (for the Italian manufactory of Vinovo).

Figure 9a illustrates an Al_2_O_3_ vs. K_2_O bivariate scatter plot of porcelain bodies. It reveals that the chemical compositions of ceramic bodies evolved over time, and that it is possible to separate distinct compositional groups. The bodies of Meissen porcelains, dated approximately between 1725 and 1782, present a high Al_2_O_3_ content (more than 32 wt%) compared to the French productions of Vieillard & Co. On the contrary, the bodies of the English and Italian porcelains have an Al_2_O_3_ content close to that of the samples from the Vieillard & Co. factory (c. 1855). Figure 9a also suggests that the compositions of productions of these different factories are quite different from each other (except for the first production of Vieillard & Co (c. 1855) which overlap English porcelains in this graph). 

At the same time, the chemical data concerning the glazes also show significant differences between the various productions compared. Figure 9b shows the bivariate CaO vs. K_2_O scatter plot of porcelain glazes. Once again, the different productions can be distinguished from each other, except for the glazes of the English productions (Bristol and Plymouth). Their compositions are close to the Saxon productions of Meissen and the first production of Vieillard & Co (c.1855) (Figure 9b). 

As a result, it seems possible to use these data in a broader context in order to document the development and experimentations in the production of porcelain in the 18th and 19th centuries in Europe. Besides, these data deserve to be completed and compared with data from other French or European porcelain factories of the 19th century.

## 5. Conclusions

To conclude, the proposed methodological approach is a first step in the characterization of this corpus of biscuits and porcelains of the Vieillard & Co manufactory. It has allowed a better understanding of this little-known production. In this paper, a group of twenty-eight archaeological sherds, which are representative of three successive production periods of the manufactory between 1851 and 1895, were investigated. The chemical composition and the microstructure of the porcelain body and the glaze were examined in order to collect information on ceramic characteristics and production technology. The analysis of the rare porcelain biscuits revealed that the support of the porcelains is made up of kaolinite clays, quartz, and potassium feldspars. Mineralogical study of the ceramic supports allowed hypotheses to be made about the temperatures of the first firing (around 950 °C) and the second firing (over 1200 °C). In the future, a study of the diffractograms by Rietveld analysis could be used to obtain more detailed evaluation of the appearance of mullite and its development in the body, but also to refine the discrimination of the productions due to the proportion of mullite and quartz [13,25,34].

The results allowed us to establish two compositional groups that differ in both the composition of the glazes and the bodies. Moreover, it is possible to distinguish the porcelain productions of the father’s and sons’ periods, thanks to both glaze and body composition analyses. Thus, it is interesting to note that significant changes in recipes and raw material supplies concur with the takeover of the factory by sons Albert and Charles. Further investigations will hopefully yield more details on the raw materials involved in the production of these long-neglected objects. Although these preliminary results contribute to answer the question of the authentication of unstamped and undecorated pieces, it should be noted that these data concern a very limited panel of French hard-paste porcelain productions of the 19th century. The chemical reference database will have to be extended to take into account other contemporary productions (such as those of Limoges or Saint-Gaudens Valentine, for example). From the perspective of a wider and more exhaustive study, the use of portable X-ray fluorescence spectrometers would be ideally suited for rapid, non-destructive, and in situ analysis of porcelain objects [35,36].

## Figures and Tables

**Table 1:** Detailed description of the investigated porcelains from the wasted dumps of the Vieillard & Co manufactory (Bordeaux, France).

Sample Number	Chronology	Manufactory	Type	Shape
BDX 20979	c. 1855	J. Vieillard & Co. (father’s production)	porcelain	bucket
BDX 20980				-
BDX 20981				plate
BDX 20982				-
BDX 20983				-
BDX 20984				-
BDX 20985				plate
BDX 20986				-
BDX 20987				-
BDX 20988				tureen lid
BDX 20989	c. 1865	J. Vieillard & Co. (sons’ production)		plate
BDX 20990				plate
BDX 20991				-
BDX 20992				-
BDX 20993				-
BDX 20994				-
BDX 20995				-
BDX 20996				-
BDX 20997				-
BDX 20998				-
BDX 20999				vase
BDX 21000				pyrometric cone
BDX 21862			biscuit of porcelain	-
BDX 21863				-
BDX 21864				-
BDX 21865				-
BDX 21866				-
BDX 21867				-
BDX 21001	c. 1885		porcelain	goblet
BDX 21002				saucer
BDX 21003				teapot
BDX 21004				insulator
BDX 21005				-
BDX 21006				plate

**Table 2:** Average chemical composition and standard deviation of the biscuit’s body, measured by EDS analysis (wt% normalized to 100%). The data are the average over at least two analyses made in different areas of the ceramic bodies; SD = standard deviation.

	Na_2_O	Al_2_O_3_	SiO_2_	K_2_O	CaO	Fe_2_O_3_
**c. 1865—J. Vieillard & Co (biscuits)**						
Mean (n = 6)	0.8	29.2	64.6	4.8	0.2	0.3
SD	0.1	1.1	0.6	0.6	0.1	0.1

**Table 3:** Average chemical composition and standard deviation of the ceramic’s body, measured by EDS analysis (wt% normalized to 100%). The data are the average over at least two analyses taken in different areas of the ceramic bodies. SD = standard deviation; nd = non detected.

	Na_2_O	MgO	Al_2_O_3_	SiO_2_	P_2_O_5_	K_2_O	CaO	TiO_2_	Fe_2_O_3_
**c. 1855—J. Vieillard & Co**									
Mean (n = 10)	2.4	0.2	24.2	68.6	0.2	3.3	0.7	nd	0.4
SD	0.3	0.1	0.7	0.7	0.1	0.8	0.2		0.1
** c. 1865—J. Vieillard & Co**									
Mean (n = 12)	1.5	0.1	29.3	64.4	0.2	3.9	0.4	0.1	0.3
SD	0.1	0.1	0.3	0.2	0.1	0.1	0.1	0.1	0.1
** c. 1885—J. Vieillard & Co**									
Mean (n = 6)	1.3	0.2	27.5	65.6	0.2	4.6	0.3	0.1	0.3
SD	0.3	0.1	2.3	2.3	0.1	0.5	0.1	0.1	0.1

**Table 4:** The average chemical composition and standard deviation of the ceramic’s body, measured by PIXE analysis (wt% normalized to 100%). The data are the average over at least two analyses taken in different areas of the ceramic bodies. SD = standard deviation; nd = non detected.

	Na_2_O	MgO	Al_2_O_3_	SiO_2_	K_2_O	CaO	TiO_2_	Fe_2_O_3_	Mn	Cu	Zn	Ga	Rb	Sr
	wt%	wt%	wt%	wt%	wt%	wt%	wt%	wt%	ppm	ppm	ppm	ppm	ppm	ppm
**c. 1855—** ** J. Vieillard & Co**														
Mean (n = 10)	1.7	0.3	24.3	68.7	3.3	0.8	0.1	0.4	78	38	69	36	197	104
SD	0.3	0.1	1.4	1.6	0.8	0.5	0.1	0.1	27	27	13	5	35	14
**c. 1865—** ** J. Vieillard & Co**														
Mean (n = 8)	1.1	nd	28.8	64.8	4.0	0.3	0.1	0.3	41	39	74	48	281	122
SD	0.1		0.3	0.4	0.1	0.1	0.1	0.1	3	5	11	3	13	7
**c. 1885—** ** J. Vieillard & Co**														
Mean (n = 6)	0.9	0.3	27.0	66.0	4.7	0.3	0.1	0.4	38	27	71	37	277	110
SD	0.2	0.1	2.3	2.3	0.6	0.1	0.1	0.1	10	11	19	10	42	19

**Table 5:** Average chemical composition and standard deviation of the glaze, measured by EDS analysis (wt% normalized to 100%). The data are the average over at least two analyses taken in different areas of the ceramic bodies. SD = standard deviation; nd = non detected.

	Na_2_O	MgO	Al_2_O_3_	SiO_2_	P_2_O_5_	K_2_O	CaO	Fe_2_O_3_
** c. 1855—J. Vieillard & Co**								
Mean (n = 10)	3.3	0.2	16.1	74.5	0.2	4.1	1.7	0.2
SD	0.5	0.1	0.6	1.0	0.1	0.9	0.3	0.1
** c. 1865—J. Vieillard & Co**								
Mean (n = 12)	1.9	nd	13.1	79.4	nd	4.8	0.7	0.2
SD	0.2		0.3	0.4		0.1	0.2	0.1
** c. 1885—J. Vieillard & Co**								
Mean (n = 6)	1.6	0.2	14.2	77.8	nd	5.5	0.5	0.3
SD	0.2	0.1	1.0	1.6		0.6	0.2	0.1

## Data Availability

All the data presented in this study are included in the Supplementary Materials; further inquiries can be directed to the corresponding author.

## Supplementary Materials

The following supporting information can be downloaded at: https://www.mdpi.com/article/10.3390/ma15155311/s1, Table S1. Chemical compositions of the bodies as determined by EDS; Table S2. Chemical compositions of the bodies as determined by PIXE-PIGE; Table S3. Chemical compositions of the glazes as determined by EDS; Table S4. Chemical compositions of the glazes as determined by PIXE-PIGE.
