# Supplementary material for: Archaeometric Characterization of the Industrial Production of Porcelains in the Vieillard & Co. Manufactory (Bordeaux, France, 19th Century)
Source: Materials (Basel). 2022 Aug 2;15(15):5311. doi: 10.3390/ma15155311 (PMC9369838; doi:10.3390/ma15155311)
Supplement: Supplementary file 1 [file materials-15-05311-s001.zip › materials-1821562-supplementary.pdf]

SUPPLEMENTARY DATA

Table S1. Chemical compositions of the bodies as determined by EDS (analyses normalised at 100 %). nd: non detected.

Sample number	Manufactory	Chronology	Na_2O	MgO	Al_2O_3	SiO_2	P_2O_5	K_2O	CaO	TiO_2	Fe_2O_3
BDX 20979	Vieillard & Co.	c. 1855	2,4	0,2	24,1	69,0	nd	3,1	0,8	nd	0,4
BDX 20980			2,8	0,2	25,3	67,2	nd	3,0	0,9	nd	0,5
BDX 20981			2,2	0,2	23,9	69,1	nd	3,5	0,7	nd	0,4
BDX 20982			2,7	0,2	23,8	69,0	nd	3,0	0,9	nd	0,4
BDX 20983			1,8	0,2	28,0	64,2	0,2	3,1	2,1	nd	0,4
BDX 20984			2,5	0,2	25,1	67,8	nd	3,1	0,7	nd	0,6
BDX 20985			1,7	nd	23,2	69,0	0,2	5,4	0,2	nd	0,3
BDX 20986			2,2	0,2	24,3	68,9	nd	3,3	0,7	nd	0,4
BDX 20987			2,7	0,2	23,6	69,4	0,2	2,7	0,9	nd	0,4
BDX 20988			2,5	0,2	24,9	68,1	0,1	3,0	0,8	nd	0,4
BDX 20989		c. 1865	1,3	nd	29,7	64,2	0,2	3,8	0,4	0,1	0,3
BDX 20990			1,6	nd	29,3	64,1	0,2	4,1	0,4	nd	0,3
BDX 20991			1,4	nd	29,4	64,4	0,2	4,0	0,3	nd	0,3
BDX 20992			1,4	nd	29,5	64,3	0,2	3,9	0,4	nd	0,3
BDX 20993			1,4	0,1	29,2	64,4	0,2	3,9	0,4	nd	0,4
BDX 20994			1,6	nd	29,2	64,5	0,2	3,9	0,3	nd	0,3
BDX 20995			1,5	nd	29,3	64,5	0,3	3,8	0,3	nd	0,3
BDX 20996			1,3	nd	29,3	64,5	0,2	3,9	0,4	0,1	0,3
BDX 20997			1,7	nd	29,0	64,4	0,2	4,0	0,4	nd	0,3
BDX 20998			1,5	nd	28,7	64,9	0,2	3,9	0,4	nd	0,3
BDX 20999			1,6	nd	29,4	64,4	0,3	3,6	0,4	nd	0,3
BDX 21000			1,5	nd	29,4	64,6	nd	3,9	0,3	nd	0,3
BDX 21862			0,9	nd	30,0	64,2	nd	4,5	0,2	nd	0,3
BDX 21863			0,9	nd	30,5	63,9	nd	4,2	0,2	nd	0,2
BDX 21864			0,8	nd	27,9	65,3	nd	5,5	0,2	nd	0,3
BDX 21865			0,8	nd	27,9	65,3	nd	5,5	0,2	nd	0,3
BDX 21866			0,8	nd	29,7	64,6	nd	4,4	0,2	nd	0,3
BDX 21867			0,8	nd	29,5	64,6	nd	4,5	0,2	nd	0,4
BDX 21001		c. 1885	1,3	0,1	29,8	63,3	0,3	4,5	0,3	nd	0,3
BDX 21002			1,7	0,1	23,2	69,4	nd	4,8	0,4	nd	0,3
BDX 21003			1,3	nd	29,2	64,0	0,2	4,6	0,4	nd	0,3
BDX 21004			0,9	0,2	27,3	67,1	0,2	3,6	0,4	0,1	0,3
BDX 21005			1,2	0,1	27,9	64,8	0,2	5,3	0,3	nd	0,4
BDX 21006			1,4	0,2	27,7	64,9	0,2	4,8	0,3	nd	0,4

Table S2. Chemical compositions of the bodies as determined by PIXE-PIGE. < ld: value below the detection limit and nd: non detected.

Sample number	Chronology	Na_2O *(wt%)*	MgO *(wt%)*	Al_2O_3 *(wt%)*	SiO_2 *(wt%)*	K_2O *(wt%)*	CaO *(wt%)*	TiO_2 *(wt%)*	Fe_2O_3 *(wt%)*	Mn *(ppm)*	Cu *(ppm)*	Zn *(ppm)*	Ga *(ppm)*	Rb *(ppm)*	Sr *(ppm)*
BDX 20979	c.1855	1,80	0,25	23,59	70,00	3,03	0,80	0,04	0,40	83	20	73	35	166	104
BDX 20980		2,03	0,33	24,81	67,97	2,94	0,83	0,06	0,57	108	36	47	38	213	100
BDX 20981		1,58	< ld	23,01	70,27	3,51	0,62	0,06	0,35	57	16	76	32	193	91
BDX 20982		1,86	< ld	23,39	69,97	2,88	0,79	0,07	0,41	92	26	72	38	166	108
BDX 20983		1,14	0,25	27,96	64,83	2,98	2,00	0,04	0,46	73	64	79	47	229	133
BDX 20984		1,72	0,29	24,73	68,44	3,11	0,65	0,05	0,54	112	14	54	40	208	79
BDX 20985		1,11	< ld	23,36	69,09	5,54	0,22	0,08	0,33	24	38	57	27	272	99
BDX 20986		1,61	0,29	24,40	68,81	3,33	0,69	0,04	0,40	58	23	62	36	194	112
BDX 20987		1,91	0,32	23,71	69,71	2,83	0,90	0,04	0,43	78	38	92	35	164	102
BDX 20988		1,76	0,33	24,39	68,92	3,11	0,80	0,04	0,40	97	103	73	36	164	108
BDX 20989	c. 1865	0,98	< ld	29,03	64,70	3,99	0,30	0,08	0,34	44	41	68	48	273	124
BDX 20990		1,14	< ld	29,10	64,16	4,19	0,29	0,08	0,33	36	47	59	47	295	136
BDX 20991		1,08	< ld	28,88	65,16	4,09	0,26	0,07	0,33	42	35	75	46	292	122
BDX 20996		0,99	< ld	28,95	65,04	4,01	0,33	0,08	0,34	40	43	61	45	275	112
BDX 20997		1,29	< ld	28,46	64,75	4,09	0,35	0,08	0,33	42	34	71	46	286	115
BDX 20998		1,06	< ld	28,34	65,68	4,07	0,36	0,05	0,33	43	35	85	49	254	125
BDX 20999		1,17	< ld	29,07	64,89	3,79	0,27	0,07	0,34	39	36	92	53	281	126
BDX 21000		1,09	< ld	28,86	64,95	4,09	0,25	0,06	0,33	38	41	79	50	289	118
BDX 21001	c. 1885	0,91	< ld	29,21	64,11	4,65	0,32	0,06	0,33	36	41	95	49	320	125
BDX 21002		1,25	< ld	22,91	69,78	4,98	0,42	0,05	0,35	40	15	86	27	258	108
BDX 21003		0,98	0,27	29,08	63,92	4,79	0,32	0,07	0,34	35	36	81	49	331	131
BDX 21004		0,71	0,26	26,45	67,62	3,66	0,39	0,15	0,37	55	13	51	34	236	81
BDX 21005		0,82	< ld	27,54	65,28	5,31	0,22	0,06	0,39	26	30	59	32	284	94
BDX 21006		1,00	0,32	27,08	65,55	5,03	0,28	0,05	0,41	34	29	52	30	235	120

Table S3. Chemical compositions of the glazes as determined by EDS (analyses normalised at 100 %). nd: non detected.

Sample number	Chronology	Manufactory	Na_2O	MgO	Al_2O_3	SiO_2	P_2O_5	K_2O	CaO	TiO_2	Fe_2O_3
BDX 20979	c. 1855	Vieillard & Co.	3,4	0,1	16,2	74,7	nd	3,7	1,6	nd	0,3
BDX 20980			3,7	nd	15,7	75,4	nd	3,6	1,3	nd	0,3
BDX 20981			3,2	nd	16,0	74,2	0,2	4,3	1,9	nd	0,2
BDX 20982			3,9	nd	16,9	73,1	nd	3,8	2,1	nd	0,2
BDX 20983			2,3	nd	16,7	74,4	nd	3,9	2,3	nd	0,3
BDX 20984			3,3	nd	15,0	76,4	nd	3,7	1,5	nd	0,2
BDX 20985			2,1	nd	16,6	73,3	nd	6,5	1,2	nd	0,3
BDX 20986			3,1	nd	16,1	74,4	0,2	4,2	1,8	nd	0,2
BDX 20987			3,6	0,1	15,7	75,0	0,2	3,5	1,7	nd	0,3
BDX 20988			3,6	nd	16,3	74,1	0,2	3,8	1,9	nd	0,2
BDX 20989	c. 1865		1,6	nd	13,0	80,1	nd	4,8	0,4	nd	0,2
BDX 20990			1,9	nd	12,9	79,6	nd	4,8	0,6	nd	0,2
BDX 20991			1,7	nd	13,0	79,3	nd	4,8	1,1	nd	0,2
BDX 20992			1,8	nd	13,2	79,5	nd	4,8	0,5	nd	0,2
BDX 20993			1,8	nd	13,6	78,7	nd	5,0	0,7	nd	0,3
BDX 20994			1,9	nd	13,1	79,4	nd	4,6	1,0	nd	nd
BDX 20995			2,0	nd	13,6	78,8	nd	4,9	0,5	nd	0,3
BDX 20996			1,7	nd	12,8	79,8	nd	4,6	0,6	nd	0,2
BDX 20997			2,1	nd	13,4	79,0	nd	4,8	0,7	nd	0,2
BDX 20998			1,9	nd	13,1	79,3	nd	4,9	0,6	nd	0,2
BDX 20999			2,2	nd	13,0	79,2	nd	4,6	0,8	nd	0,2
BDX 21000			1,8	nd	12,8	79,8	nd	4,8	0,7	nd	0,2
BDX 21001	c. 1885		1,6	nd	13,1	79,4	nd	5,3	0,4	nd	0,2
BDX 21002			2,0	0,1	14,5	77,3	nd	5,4	0,6	nd	0,3
BDX 21003			1,5	nd	13,5	78,9	nd	5,6	0,3	nd	0,3
BDX 21004			1,4	0,1	13,7	78,8	nd	4,8	0,9	nd	0,3
BDX 21005			1,5	nd	15,8	75,4	nd	6,4	0,5	nd	0,4
BDX 21006			1,7	nd	15,0	76,7	nd	5,9	0,5	nd	0,2

Table S4. Chemical compositions of the glazes as determined by PIXE-PIGE. < ld: value below the detection limit and nd: non detected.

Sample number	Chronology	Na_2O *(wt%)*	MgO *(wt%)*	Al_2O_3 *(wt%)*	SiO_2 *(wt%)*	P_2O_5 *(wt%)*	K_2O *(wt%)*	CaO *(wt%)*	TiO_2 *(wt%)*	Fe_2O_3 *(wt%)*	Mn *(ppm)*	Ni *(ppm)*	Cu *(ppm)*	Zn *(ppm)*	Ga *(ppm)*	Rb *(ppm)*	Sr *(ppm)*	Ba *(ppm)*
BDX 20979	c.1855	3,06	< ld	17,26	73,20	< ld	4,19	1,75	< ld	0,44	91	248	36	58	30	274	314	680
BDX 20980		3,34	< ld	16,86	74,09	< ld	3,83	1,36	0,04	0,44	72	190	50	14	26	233	208	679
BDX 20981		2,94	0,08	18,81	70,87	0,09	4,67	2,42	< ld	0,25	39	113	33	41	21	223	302	473
BDX 20982		3,61	< ld	17,59	72,08	0,11	3,98	2,48	< ld	0,22	30	154	28	35	21	194	300	504
BDX 20984		3,13	< ld	17,43	73,38	< ld	4,13	1,70	< ld	0,26	42	154	26	23	22	223	242	413
BDX 20985		1,82	< ld	17,20	72,37	< ld	7,00	1,27	< ld	0,36	36	146	28	30	39	330	242	369
BDX 20986		2,69	< ld	17,98	72,22	< ld	4,72	2,04	< ld	0,31	39	103	50	68	24	224	285	496
BDX 20987		3,26	< ld	16,94	73,66	< ld	3,81	1,99	< ld	0,34	43	126	29	66	23	188	267	424
BDX 20988		3,48	0,10	18,15	71,41	0,09	4,11	2,25	0,04	0,47	78	164	30	74	24	239	298	757
BDX 20989		1,72	< ld	13,00	79,53	0,17	4,84	0,40	0,04	0,34	34	26	46	15	7	354	155	540
BDX 20990	c. 1865	1,37	< ld	12,06	81,17	< ld	4,54	0,34	0,04	0,51	22	99	41	29	24	302	123	670
BDX 20991		1,61	< ld	14,09	77,92	0,44	4,83	1,01	< ld	0,17	24	6	40	9	10	218	217	1018
BDX 20992		1,38	< ld	12,94	80,88	< ld	4,39	0,28	< ld	0,20	23	20	45	40	25	305	100	478
BDX 20993		1,50	< ld	12,63	80,50	< ld	4,65	0,42	0,02	0,33	25	39	39	48	21	283	120	641
BDX 20994		1,62	< ld	15,33	75,97	0,45	5,07	1,37	< ld	0,21	29	20	48	12	12	236	298	1334
BDX 20995		1,78	< ld	14,11	77,81	0,08	5,47	0,52	< ld	0,32	36	44	47	34	22	352	148	528
BDX 20996		1,69	< ld	14,25	77,48	< ld	5,45	0,85	< ld	0,27	38	52	55	23	24	338	217	978
BDX 20997		1,69	< ld	13,91	77,87	0,10	5,27	0,98	< ld	0,21	27	31	45	28	15	302	226	1070
BDX 20998	c. 1885	1,80	< ld	13,84	78,44	< ld	5,09	0,61	< ld	0,21	26	20	46	33	22	307	183	958
BDX 20999		1,63	< ld	15,17	76,47	< ld	5,43	1,03	< ld	0,19	27	73	55	21	23	275	220	1157
BDX 21000		1,66	< ld	14,29	77,81	0,12	5,14	0,81	< ld	0,20	20	28	50	35	16	292	205	1050
BDX 21001		1,44	< ld	14,34	77,07	< ld	6,13	0,56	< ld	0,25	32	27	52	49	23	371	170	2169
BDX 21002		1,96	< ld	16,36	74,40	0,10	5,98	0,91	0,03	0,35	60	99	24	30	22	264	152	552
BDX 21003		1,52	< ld	13,72	77,50	< ld	6,28	0,53	0,03	0,39	43	14	56	52	24	494	170	651
BDX 21004		1,33	0,15	14,03	77,34	0,09	5,45	1,02	< ld	0,61	80	48	33	42	23	368	234	911
BDX 21005		1,32	< ld	15,54	75,69	< ld	6,55	0,53	0,02	0,41	34	102	26	16	21	285	145	351
BDX 21006		1,54	< ld	15,30	76,23	< ld	6,21	0,47	0,02	0,28	28	23	35	19	30	296	130	373
